# Supplementary material for: Nutraceutical Potential of Lentinula edodes’ Spent Mushroom Substrate: A Comprehensive Study on Phenolic Composition, Antioxidant Activity, and Antibacterial Effects
Source: J Fungi (Basel). 2023 Dec 15;9(12):1200. doi: 10.3390/jof9121200 (PMC10744564; doi:10.3390/jof9121200)
Supplement: Supplementary file 1 [file jof-09-01200-s001.zip › jof-2685579-supplementary.pdf]

Figure S1. Antibacterial assay *S. aureus* (ATCC 23235) example of the antagonistic effect of the extracts on the antibiotic used.

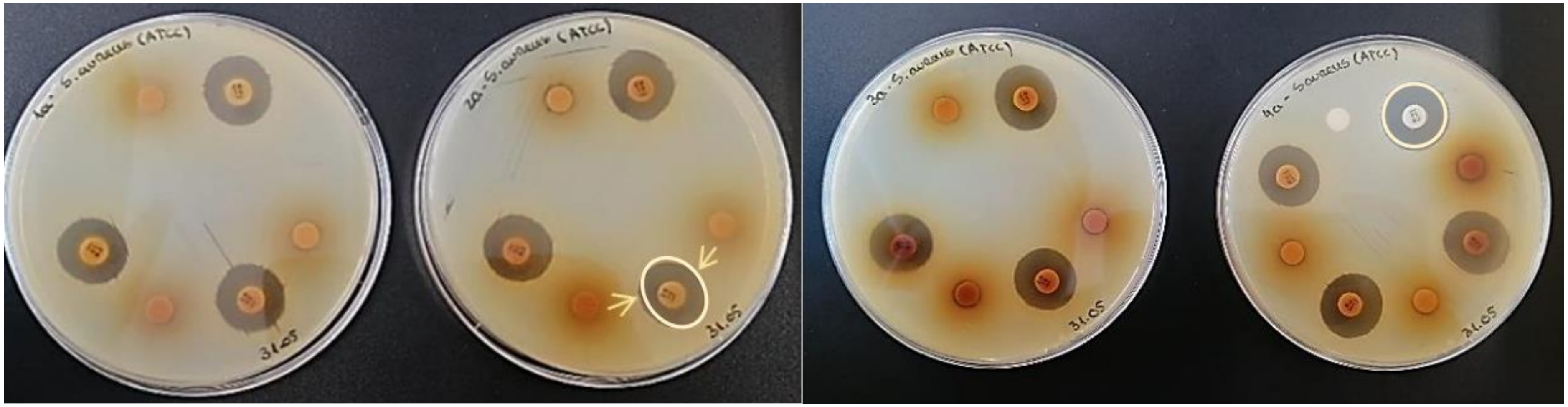

Table S1. Polyphenol Composition Analysis in Spent Mushroom Substrate Extracts: Impact of Solvent and Extraction Method

Samples	Total Phenols (mg GA g^{-1})	*Ortho*-diphenols (mg GA g^{-1})	Flavonoids (mg CAT g^{-1})
Solvent			
w	97.60 ± 2.46^{b}	319.93 ± 4.26^{b}	40.20 ± 1.35^{b}
et	156.98 ± 2.13^{a}	395.74 ± 4.41^{a}	57.53 ± 1.35^{a}
Extraction method			
SE	103.10 ± 4.12^{cd}	242.80 ± 7.63^{e}	242.80 ± 7.63^{c}
LE	98.52 ± 3.69^{d}	260.89 ± 6.83^{e}	260.89 ± 6.83^{c}
LE-HT	190.61 ± 4.12^{a}	517.07 ± 7.63^{a}	517.07 ± 7.63^{a}
UE	111.37 ± 3.69^{c}	347.17 ± 8.36^{c}	347.17 ± 8.36^{b}
COMB	103.52 ± 4.12^{cd}	323.16 ± 6.83^{d}	323.16 ± 6.83^{b}
COMB-HT	156.63 ± 4.12^{b}	455.91 ± 7.63^{b}	455.91 ± 7.63^{a}

w, distilled water; et, ethanol 50% (v/v) solvent; SE, short extraction; LE, 24 h extraction; LE-HT, 24 h extraction with high temperature; UE, ultrasound extraction; COMB, combined methods (LE+UE); COMB-HT, combined methods (LE-HT+UE). The presented values are means ± standard deviation of triplicate measurements. Means in the same column with different letters were significantly different ($P<0.05$, ANOVA, Tukey-HSD).

Table S2. Results of the evaluation of the antioxidant capacity of the spent mushroom substrate extracts, by solvent and by extraction method.

Samples	ABTS$^{•+}$ (mmol Trolox g^{-1})	DPPH$^{•}$ (mmol Trolox g^{-1})	FRAP (mmol Trolox g^{-1})
Solvent			
w	0.487 ± 0.010^{b}	0.327 ± 0.008^{b}	0.675 ± 0.012^{a}
et	0.665 ± 0.011^{a}	0.777 ± 0.007^{a}	0.696 ± 0.012^{a}
Extraction method			
SE	0.409 ± 0.017^{c}	0.525 ± 0.013^{c}	0.570 ± 0.020^{c}
LE	0.510 ± 0.017^{b}	0.463 ± 0.013^{d}	0.573 ± 0.022^{c}
LE-HT	0.762 ± 0.021^{a}	0.855 ± 0.014^{a}	1.031 ± 0.020^{a}
UE	0.533 ± 0.019^{b}	0.411 ± 0.014^{e}	0.563 ± 0.025^{c}
COMB	0.538 ± 0.017^{b}	0.475 ± 0.014^{d}	0.521 ± 0.020^{c}
COMB-HT	0.703 ± 0.019^{a}	0.584 ± 0.014^{b}	0.857 ± 0.020^{b}

w, distilled water; et, ethanol 50% (v/v) solvent; SE, short extraction; LE, 24 h extraction; LE-HT, 24 h extraction with high temperature; UE, ultrasound extraction; COMB, combined methods (LE+UE); COMB-HT, combined methods (LE-HT+UE). The presented values are means ± standard deviation of triplicate measurements. Means in the same column with different letters were significantly different ($P<0.05$, ANOVA, Tukey-HSD).

Table S3. Diameter measurements for each extract alone compared with the positive control (gentamicin).

	S. aureus (ATCC 23235)	*A. hydrophila* (C2GSPA1)	*P. aeruginosa* (C3GSPR1)	*V. fluvialis* (RimA1TCBS)
Control (+)	18.0[a]	18.0[a]	12.0[a]	23.0[a]
w-SE	0.0[c]	8.0[e]	8.0[d]	7.0[c]
et-SE	7.0[b]	0.0[j]	9.0[c]	0.0[d]
w-LE	0.0[c]	9.0[d]	9.0[c]	7.0[c]
et-LE	7.0[b]	8.0[e]	8.0[d]	0.0[d]
w-LE-HT	0.0[c]	9.0[c]	9.0[c]	8.0[b]
et-LE-HT	7.0[b]	10.0[b]	10.0[b]	7.0[c]
w-UE	7.0[b]	8.0[f]	8.0[d]	0.0[d]
et-UE	0.0[c]	0.0[j]	9.0[c]	0.0[d]
w-COMB	0.0[c]	7.0[g]	8.0[d]	0.0[d]
et-COMB	0.0[c]	0.0[i]	7.0[f]	0.0[d]
w-COMB-HT	0.0[c]	8.0[f]	8.0[d]	0.0[d]
et-COMB-HT	7.0[b]	7.0[h]	8.0[e]	7.0[c]

Means (n=2) in the same column with different letters were significantly different ($P<0.05$, ANOVA, t-student). Caption: “w” distilled water, “et” ethanol 50% (v/v) solvent, “SE” short extraction, “LE” 24h extraction, “LE-HT” 24h extraction with high temperature, “UE” ultrasound extraction, “COMB” combined methods (LE+UE), “COMB-HT” combined methods (LE-HT+UE).

Table S4. Halo measurements for the extracts according to solvent and extraction method used.

	S. aureus (ATCC 23235)	*A. hydrophila* (C2GSPA1)	*P. aeruginosa* (C3GSPR1)	*V. fluvialis* (RimA1TCBS)
Solvent				
w	1.17^{b}	8.17^{a}	$8,17^{b}$	3.67^{a}
Et:H_20	4.67^{a}	4.17^{b}	8.67^{a}	2.33^{b}
Method				
SE	3.5^{a}	4.0^{d}	8.5^{b}	3.5^{b}
LE	3.5^{a}	8.5^{b}	8.5^{b}	3.5^{b}
LE-HT	3.5^{a}	9.5^{a}	9.5^{a}	7.5^{a}
UE	3.5^{a}	4.0^{d}	8.5^{b}	0.0^{c}
COMB	0.0^{b}	3.5^{e}	7.5^{d}	0.0^{c}
COMB-HT	3.5^{a}	7.5^{c}	8.0^{c}	3.5^{b}

Means (n=2) in the same column with different letters were significantly different ($P<0.05$, ANOVA, t-student). Caption: "w" distilled water, "et" ethanol 50% (v/v) solvent, "SE" short extraction, "LE" 24h extraction, "LE-HT" 24h extraction with high temperature, "UE" ultrasound extraction, "COMB" combined methods (LE+UE), "COMB-HT" combined methods (LE-HT+UE).

Table S5. Halo measurements for the extracts combined with gentamicin (CN10) compared with the positive control (gentamicin).

	S. aureus (ATCC 23235)	*S. aureus* (C511)	*S. aureus* (C612)	*E. faecium* (C1)	*E. faecium* (C14)	*E. coli* (ATCC 25922)	*A. hydrophila* (C2GSPA1)	*P. aeruginosa* (C3GSPR1)	*V. fluvialis* (RimA1TCBS)
Control (+)	18.0^{b}	15.0^{b}	17.0^{a}	17.0^{ab}	16.0^{c}	16.0^{a}	18.0^{a}	12.0^{c}	23.0^{a}
w-SE	16.0^{d}	13.0^{d}	16.5^{ab}	15.0^{d}	15.0^{d}	0.0^{j}	16.0^{c}	9.0^{e}	20.0^{c}
et-SE	17.0^{c}	13.0^{d}	15.5^{b}	15.0^{d}	15.0^{d}	14.0^{d}	16.0^{c}	10.0^{d}	20.0^{d}
w-LE	16.0^{d}	14.0^{c}	16.5^{ab}	16.0^{c}	16.0^{c}	0.0^{j}	17.0^{b}	9.0^{e}	20.0^{d}
et-LE	17.0^{c}	13.0^{d}	16.5^{ab}	16.5^{bc}	16.5^{bc}	14.0^{c}	15.0^{d}	10.0^{d}	21.0^{b}
w-LE-HT	17.0^{c}	15.0^{b}	17.0^{a}	16.0^{c}	16.0^{c}	7.0^{i}	18.0^{a}	13.0^{a}	20.0^{d}
et-LE-HT	16.0^{d}	14.0^{c}	16.5^{ab}	16.0^{c}	16.0^{c}	12.0^{f}	14.0^{e}	9.0^{e}	20.0^{d}
w-UE	17.0^{c}	14.0^{c}	16.5^{ab}	16.0^{c}	16.0^{c}	16.0^{a}	16.0^{c}	9.0^{e}	20.0^{d}
et-UE	17.0^{c}	13.0^{d}	16.0^{ab}	17.0^{ab}	17.0^{ab}	15.0^{b}	15.0^{d}	9.0^{e}	20.0^{d}
w-COMB	16.0^{d}	14.0^{c}	16.5^{ab}	16.5^{bc}	16.5^{bc}	13.0^{e}	16.0^{c}	9.0^{e}	20.0^{d}
et-COMB	17.0^{c}	13.0^{d}	16.0^{ab}	16.0^{c}	16.0^{c}	12.0^{g}	15.0^{d}	9.0^{e}	19.0^{e}
w-COMB-HT	17.0^{c}	14.0^{c}	16.5^{ab}	16.5^{bc}	16.5^{bc}	13.0^{e}	16.0^{c}	9.0^{e}	20.0^{d}
et-COMB-HT	18.0^{a}	15.0^{a}	16.5^{ab}	17.5^{a}	17.5^{a}	9.0^{h}	16.0^{c}	12.0^{b}	20.0^{d}

Means (n=2) in the same column with different letters were significantly different (P<0.05, ANOVA, t-student). Caption: “w” distilled water, “et” ethanol 50% (v/v) solvent, “SE” short extraction, “LE” 24h extraction, “LE-HT” 24h extraction with high temperature, “UE” ultrasound extraction, “COMB” combined methods (LE+UE), “COMB-HT” combined methods (LE-HT+UE).

Table S6. Halo measurements for the extracts combined with gentamicin according to solvent and extraction method used.

	S. aureus (ATCC 23235)	*S. aureus* (C511)	*S. aureus* (C612)	*E. faecium* (C1)	*E. faecium* (C14)	*E. coli* (ATCC 25922)	*A. hydrophila* (C2GSPA1)	*P. aeruginosa* (C3GSPR1)	*V. fluvialis* (RimA1TCBS)
Solvent									
H_20	16.50^{b}	14.00^{a}	16.58^{a}	16.00^{a}	16.00^{a}	8.17^{b}	16.50^{a}	9.67^{b}	20.08^{a}
Et:H_20	17.00^{a}	13.50^{b}	$16,17^{a}$	16.33^{a}	16.33^{a}	12.67^{a}	15.17^{b}	9.83^{a}	20.00^{a}
Method									
SE	16.50^{c}	13.00^{c}	16.00^{a}	15.00^{c}	15.00^{c}	7.00^{e}	16.00^{a}	9.50^{c}	20.25^{ab}
LE	16.50^{c}	13.50^{b}	16.50^{a}	16.25^{b}	16.25^{b}	7.00^{e}	16.00^{a}	9.50^{c}	20.50^{a}
LE-HT	16.50^{c}	14.50^{a}	16.75^{a}	16.00^{b}	16.00^{b}	9.50^{d}	16.00^{a}	11.00^{a}	20.00^{b}
UE	17.00^{b}	13.50^{b}	16.25^{a}	16.50^{ab}	16.50^{ab}	15.50^{a}	15.50^{b}	9.00^{d}	20.00^{b}
COMB	16.50^{c}	13.50^{b}	16.25^{a}	16.25^{b}	16.25^{b}	12.50^{b}	15.50^{b}	9.00^{d}	19.50^{c}
COMB-HT	17.50^{a}	14.50^{a}	16.50^{a}	17.00^{a}	17.00^{a}	11.00^{c}	16.00^{a}	10.50^{b}	20.00^{b}

Means (n=2) in the same column with different letters were significantly different ($P<0.05$, ANOVA, t-student). Caption: "w" distilled water, "et" ethanol 50% (v/v) solvent, "SE" short extraction, "LE" 24h extraction, "LE-HT" 24h extraction with high temperature, "UE" ultrasound extraction, "COMB" combined methods (LE+UE), "COMB-HT" combined methods (LE-HT+UE).
